# Specific Expression of Channelrhodopsin-2 in Single Neurons of *Caenorhabditis elegans*

**DOI:** 10.1371/journal.pone.0043164

**Published:** 2012-08-30

**Authors:** Cornelia Schmitt, Christian Schultheis, Steven J. Husson, Jana F. Liewald, Alexander Gottschalk

**Affiliations:** 1 Buchmann Institute for Molecular Life Sciences, Goethe-University, Frankfurt, Germany; 2 Institute of Biochemistry, Goethe-University, Frankfurt, Germany; 3 Functional Genomics and Proteomics, Katholieke Universiteit, Leuven, Belgium; Brown University, United States of America

## Abstract

Optogenetic approaches using light-activated proteins like Channelrhodopsin-2 (ChR2) enable investigating the function of populations of neurons in live *Caenorhabditis elegans* (and other) animals, as ChR2 expression can be targeted to these cells using specific promoters. Sub-populations of these neurons, or even single cells, can be further addressed by restricting the illumination to the cell of interest. However, this is technically demanding, particularly in free moving animals. Thus, it would be helpful if expression of ChR2 could be restricted to single neurons or neuron pairs, as even wide-field illumination would photostimulate only this particular cell. To this end we adopted the use of Cre or FLP recombinases and conditional ChR2 expression at the intersection of two promoter expression domains, i.e. in the cell of interest only. Success of this method depends on precise knowledge of the individual promoters' expression patterns and on relative expression levels of recombinase and ChR2. A bicistronic expression cassette with GFP helps to identify the correct expression pattern. Here we show specific expression in the AVA reverse command neurons and the aversive polymodal sensory ASH neurons. This approach shall enable to generate strains for optogenetic manipulation of each of the 302 *C. elegans* neurons. This may eventually allow to model the *C. elegans* nervous system in its entirety, based on functional data for each neuron.

## Introduction

Optogenetic approaches to control cellular activity are increasingly used in the neurosciences, to decipher the function of neuronal populations within neuronal circuits or to precisely control synaptic transmission and/or plasticity [1]–[5]. Several optogenetic tools have been established or generated to date. These include channelrhodopsins and variants thereof, which are light-gated cation channels allowing to photodepolarize the membrane and to activate cells [1], [2], [6], [7]. Halorhodopsin (NpHR) [8], a light driven chloride importer, and outward directed proton pumps (Arch and Mac) [9], are used for photohyperpolarization and thus inactivation of cells. Also light-activated enzymes like photoactivated adenylate cyclase (PAC) [10]–[12] to stimulate intracellular 2^nd^ messenger signaling, photoswitchable protein tags like the LOV domain or phototriggered protein-protein interaction modules are used [13], [14]. These proteins are generally expressed using cell-type specific promoters, e.g. those of vesicular acetylcholine- or GABA transporters, to restrict them to certain neuronal populations [4]. Further specificity of cell manipulation may be achieved by selective illumination of the cell of interest, however, this can be technically demanding [15], [16]. Thus, expression of the optogenetic switch in single cells would be highly beneficial, as wide-field illumination would still just activate the cell of interest. In few cases in *C. elegans*, single-cell specific promoters have been described that may be employed, but these are rare, and their utility can be limited as the achievable expression levels may be too low. A more generic way to achieve selective expression at high levels is thus needed. This could even enable “functional mapping” of the *C. elegans* nervous system in a neuron-by-neuron manner.

**Figure 1:**
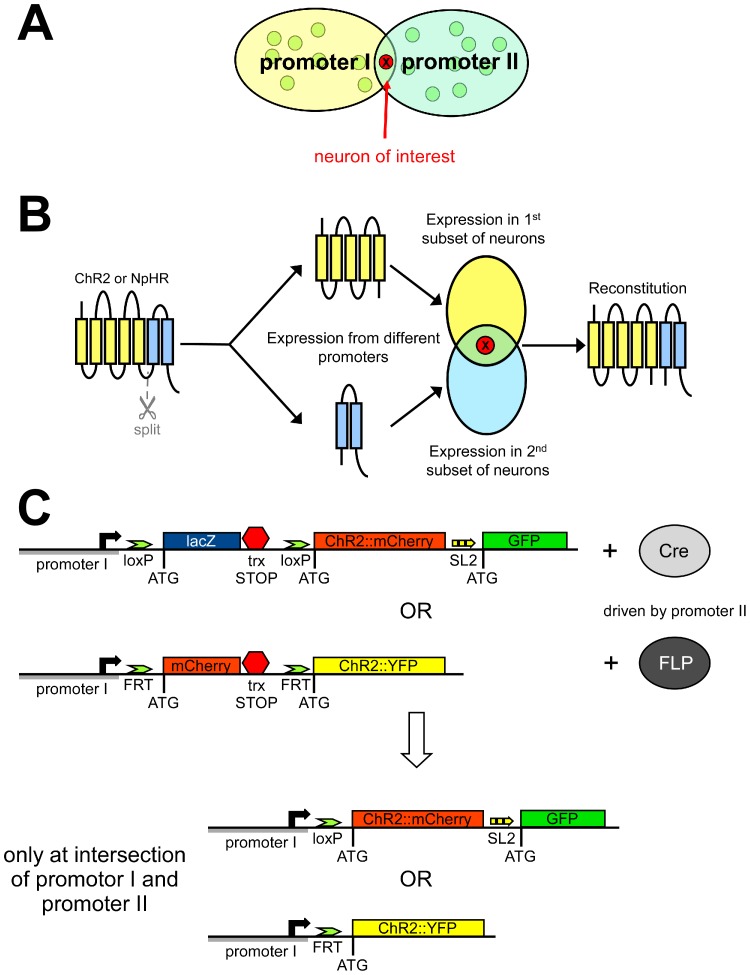
Conditional expression of ChR2 in single neurons using two promoters of intersecting expression domain, and DNA recombinases. **A**) Two promoters, each expressing in a different set of neurons, with overlapping expression in one neuron only. **B**) Concept of achieving conditional expression using split proteins that can reconstitute functional ChR2 or NpHR in the neuron of interest. See also Fig. S1. **C**) Conditional expression is achieved by encoding ChR2 form a construct (driven by promoter I) that is blocked by a transcriptional stop cassette (red hexagon) between promoter and ChR2 start codon, and is flanked by recombinase recognition sites (loxP or FRT sites, recognized by Cre or FLP recombinases, respectively). The respective recombinase is driven by promoter II, to generate a transcription-competent DNA construct encoding ChR2 and (optionally) soluble GFP from a bicistronic expression cassette (*via* SL2 trans-splicing).

Currently, two main approaches to specifically express proteins in single cells of *C. elegans* have been used, both having in common the use of two promoters with coinciding expression in just the cell of interest (**Fig. 1A**): In the first approach, two protein fragments of the protein of interest are encoded by constructs driven by each of the two promoters, and reconstitute a functional protein when co-expressed (**Fig. 1B**) [17], [18]. As there was precedent for functional reconstitution of bacteriorhodopsin from protein fragments [19], we attempted to achieve this goal for ChR2 and NpHR, by splitting the proteins in loops between transmembrane helices, and attaching leucine zippers and/or split GFP fragments to the new termini to enhance reconstitution; however, despite testing numerous split sites, rhodopsin function after in *vivo* reconstitution was too low to be useful (**Fig. S1**). The second approach (**Fig. 1C**) uses genetic techniques, where the construct encoding the protein of interest is conditionally expressed only when a recombinase removes a transcriptional stop cassette, flanked by recognition sites for either FLP or Cre recombinase, which prevents expression of the respective protein. As two promoters are used for the two constructs, expression is thus found only at the intersection of both promoter expression domains (**Fig. 1C**). Both expression systems have been established for *C. elegans* [20], [21], and one publication already demonstrated the application of the FLP system for ChR2 expression in the neuron pair ASH [22].

Cre is a recombinase from the bacteriophage P1 [23], recognizing 34 bp DNA sequences termed loxP sites, that removes or inverts the DNA between them, depending on the orientation of the loxP sites (**Fig. 1C**) [24]. Cre is a commonly used tool for both in *vitro* and in *vivo* gene manipulation [25]. The FLP system follows the same basic principle. FLP recombinase recognizes FRT sites in the DNA flanking the sequence to be excised (**Fig. 1C**). Davis *et al.* used this to achieve GFP expression cell-specifically [21]. Their stop cassette contained a red fluorescent marker (mCherry), thus allowing to follow the expression pattern of the “off” state expression cassette; the mCherry coding sequence is removed, i.e. expression abrogated, when FLP activates the expression cassette. Davis *et al.* [21] generated constructs based on the Gateway system, which adds some extra sequence in the reading frame. As these sequences are also translated, they may cause problems in the final protein.

The main focus of our work is on two pairs of neurons involved in evoking or generating the backward escape response: ASH and AVA neurons. Cell bodies of both neuron types are localized in the nerve ring ganglia in the head [26]. The pair of ASH sensory neurons extends ciliated dendrites to the nose of the animal. ASH detects aversive stimuli to the head, including touch, nociceptive chemicals and osmotic pressure [27]. AVA neurons are command interneurons that integrate signals from several types of sensory neurons, mainly in the anterior part of the animal, and among the other backward command neurons (AVD, AVE) is classified as the most potent inducer of backward locomotion [28], [29]. Here, we demonstrate expression of ChR2 in AVA backward command neurons and ASH polymodal sensory neurons, using and comparing both Cre and FLP recombinase systems. We further attempted to express ChR2 in the pair of PVC tail neurons, which are command interneurons leading to a forward movement [28], [30]. While in principle straightforward, the approach can require a significant amount of empirical optimization. Here we describe how single-cell expression of ChR2 can be achieved using either Cre or FLP recombinases, and which critical points need to be considered. The long-term goal could be to generate, in a joint effort by many labs, a collection of strains expressing ChR2 and/or other optogenetic tools in as many single neurons as possible.

## Materials and Methods

### Strains

*C. elegans* strains were cultivated on nematode growth medium (NGM) with the *E. coli* strain OP50-1. The following genetic backgrounds were used: N2 (wild type), *lite-1(ce314)*, *lin-15(n765ts); lite-1(ce314)*. We also used the transgenic strain AQ2334: *lite-1(ce314); ljIs123[pmec-4::ChR2(codon optimized); Punc-122::rfp]*, a kind gift of W. Schafer (MRC Laboratory of Molecular Biology, Cambridge, UK). To create the transgenic ChR2 expression worm lines the plasmids described below were injected into the gonads of hermaphrodite animals [31]. Plasmids containing the recombinase recognition site-flanked ChR2 and plasmids containing the recombinase were co-injected, at concentrations listed below. In one case we integrated the extrachromosomal array using trimethyl-oxalen (Sigma Aldrich) and irradiation with 365 nm UV light [22].

These transgenic strains were prepared: **ZX1019**: *lin-15(n765ts); lite-1(ce314); zxEx716[pglr-1::loxP::LacZ::STOP::loxP::ChR2::mCherry::SL2::GFP* (80 ng/µl); *pgpa-14::Cre* (80 ng/µl); *lin-15+]*, **ZX1020**: *lin-15(n765ts); lite-1(ce314); zxEx704[pflp18::loxP::LacZ::STOP::loxP::ChR2::mCherry::SL2::GFP* (80 ng/µl); *pgpa-14::Cre* (80 ng/µl); *lin-15+]*, **ZX1021**: *lin-15(n765ts); lite-1(ce314); zxEx705[posm-10::loxP::LacZ::STOP::loxP::ChR2::mCherry:: SL2::GFP* (80 ng/µl); *pgpa-11::Cre* (80 ng/µl); *lin-15+]*, **ZX1023**: *lin-15(n765ts); lite-1(ce314); zxIs30[pflp-18::loxP::LacZ::STOP::loxP::ChR2::mCherry::SL2::GFP* (80 ng/µl); *pgpa-14::Cre* (80 ng/µl); *lin-15+]*, **ZX1084**: *lin-15(n765ts); lite-1(ce314); zxEx707[p-flp18::loxP::LacZ::STOP::loxP::ChR2::mCherry::SL2::GFP* (80 ng/µl); *prig-3::Cre* (80 ng/µl); *lin-15+]*, **ZX1085**: *lin-15(n765ts); lite-1(ce314); zxEx711[posm-10::loxP::LacZ::STOP::loxP::ChR2::mCherry:: SL2::GFP* (80 ng/µl); *pnhr-79::Cre* (150 ng/µl); *lin-15+]*, **ZX1379**: *lin-15(n765ts); lite-1(ce314); zxEx715[psra-6::loxP::LacZ::STOP::loxP::ChR2::mCherry::SL2::GFP* (150 ng/µl); *pnhr-79::Cre* (150 ng/µl); *lin-15+]*, **ZX1380**: *lin-15(n765ts); zxEx717[pflp-18::FRT::mCherry::STOP::FRT::ChR2YFP* (80 ng/µl); *prig-3::FLP* (80 ng/µl); *lin-15+]*, **ZX1394**: *N2; zxEx718[pglr-1::loxP::LacZ::STOP::loxP::ChR2::mCherry* (80 ng/µl); *pdes-2::Cre* (80 ng/µl)], **ZX1395**: *N2; zxEx719 [pnmr-1::loxP::LacZ:: STOP::loxP::ChR2::mCherry* (80 ng/µl); *pdes-2::Cre* (80 ng/µl)], N2; zxEx [pCS1 (100 ng/µl), pCS2 (100 ng/µl); rol-6d (80 ng/µl)], N2; zxEx [pCS1+ (20 ng/µl), pCS2 (20 ng/µl); rol-6d (80 ng/µl)], N2; zxEx [pCS1+ (100 ng/µl), pCS6 (100 ng/µl); rol-6d (80 ng/µl)], N2; zxEx [pCS3 (100 ng/µl), pCS4 (100 ng/µl); rol-6d (80 ng/µl)], N2; zxEx [pCS5 (50 ng/µl), pCS6 (50 ng/µl); rol-6d (80 ng/µl)], N2; zxEx [pCS10 (50 ng/µl); rol-6d (80 ng/µl)]

N2; zxEx [pCS14 (100 ng/µl), pCS16 (100 ng/µl); rol-6d (80 ng/µl)], N2; zxEx [pCS14+ (100 ng/µl), pCS16 (100 ng/µl); rol-6d (80 ng/µl)], N2; zxEx [pAG54 (100 ng/µl); rol-6d (80 ng/µl)]

N2; zxEx [pmyo-3::NpHR::eCFP (50 ng/µl); rol-6d (80 ng/µl)], *lin-15(n765ts)*; zxEx [pCS20 (50 ng/µl), pCS23 (50 ng/µl); lin-15+ (80 ng/µl)], *lin-15(n765ts)*; zxEx [pCS21 (50 ng/µl), pCS24 (50 ng/µl); lin-15+ (80 ng/µl)], *lin-15(n765ts)*; zxEx [pCS21 (50 ng/µl), pCS24+ (50 ng/µl); lin-15+ (80 ng/µl)], *lin-15(n765ts)*; zxEx [pCS22 (50 ng/µl), pCS25 (50 ng/µl); lin-15+ (80 ng/µl)]

*lin-15(n765ts)*; zxEx [pCS26 (50 ng/µl); lin-15+ (80 ng/µl)], *lin-15(n765ts)*; zxEx [pCS27 (50 ng/µl); lin-15+ (80 ng/µl)], *lin-15(n765ts)*; zxEx [pCS28 (50 ng/µl); lin-15+ (80 ng/µl)]

*lin-15(n765ts)*; zxEx [pCS83 (50 ng/µl), pCS85 (50 ng/µl); lin-15+ (80 ng/µl)], *lin-15(n765ts)*; zxEx [pCS80 (50 ng/µl), pCS82 (50 ng/µl); lin-15+ (80 ng/µl)], *lin-15(n765ts)*; zxEx [pCS81 (50 ng/µl), pCS82 (50 ng/µl); lin-15+ (80 ng/µl)], *lin-15(n765ts)*; zxEx [pCS89+ (50 ng/µl), pCS90+ (50 ng/µl); lin-15+ (80 ng/µl)], *lin-15(n765ts)*; zxEx [pCS89+ (50 ng/µl), pCS91+ (50 ng/µl); lin-15+ (80 ng/µl)], *lin-15(n765ts)*; zxEx [pCS92 (50 ng/µl), pCS94 (50 ng/µl); lin-15+ (80 ng/µl)]

### Plasmids

The following plasmids were kindly provided by N. Pokala (Bargmann lab, Rockefeller University, USA): pNP165: P*glr-1*::flox::ChR2::mCherry, pNP259: P*gpa-14*::Cre, pNP260: P*nmr-1*::flox::ChR2::mCherry. Plasmids pWD172 (Entry-Vector for Slot-2 in Gateway cloning containing FLP) and pWD178 (Entry-Vector for Slot-2 in Gateway cloning containing FRT::mCherry::STOP::FRT) were kindly provided by E. Jorgensen [21]. The plasmid pTNZ126 (containing FLP::unc-54-UTR) was a kind gift from W. Schafer. In addition, these plasmids were kindly provided: **TU#712** (*nzYFP = YFP(aa1-157)::zipper*); **TU#715** (*czCFP = zipper::CFP(aa155-239)*) (gifts by M. Chalfie [17]); ***spGFP1-10*** *(GFP(aa1-214));* ***spGFP11*** *(pat-3-signalpeptide::GFP(aa215-230))* (gifts by C. Bargmann [32]). Plasmids **pAG54** (p*myo-3*::ChR2::YFP) and p*myo-3*::NpHR::eCFP were described previously [1], [8].

The following plasmids were prepared in this work: pCoS2 (p*nhr-79*::Cre): As backbone, plasmid pNP259, cut with SphI and XmaI, was used. The inserted *nhr-79* promoter was amplified from genomic DNA using oligos CAAGCTTGCATGCGCGGATAGACT TCCAGTTGTGAAT and CCATGGTACCGTCGATTTTATGCTAAAAATCGATAAATCAAGG. The insert was cloned using the In-Fusion Cloning Kit (CloneTech, USA). pCoS3 (p*sra-6*::loxP::LacZ::STOP::loxP::ChR2::mCherry): As backbone, plasmid pNP165, cut with FseI and AscI, was used. The *sra-6* promoter was amplified from p*sra-6*::ChR2::YFP using oligos GCGGCCAAACATGATCTTAC and CATACCTTTGGGTCCTTTGG. pCoS6 (p*glr-1*::loxP::LacZ::STOP::loxP::ChR2::mCherry::SL2::GFP): To insert coding sequence for bicistronically expressed GFP into plasmid pNP165, an AvrII restriction site was added after ChR2::mCherry by site-directed mutagenesis; then the plasmid was opened using AvrII. The insert for bicistronically expressed GFP was amplified from plasmid pEntry(policys)GFP (a gift from M. de Bono, MRC Laboratory of Molecular Biology, Cambridge, UK) using oligos GGACCCAAAGGTATGTTTCG and TTAGGTACTAGTCGCTCAGTTGGAATTCTACG. pCoS10 (p*flp-18*::loxP::LacZ::STOP::loxP::ChR2::mCherry::SL2::GFP): pCoS6 was cut with SphI and BamHI, then the *flp-18* promoter was inserted after amplification from plasmid pCS40 using oligos AAGCTTGGCCGGCCTCTGTCACATACTGCTCGAATC and AAGCTT GGCGCGCCGTCTAACCCTGAAATTATTATTTTTAGTTG. pCoS11 (p*rig-3*::Cre): Plasmid pNP259 was cut with SphI and XmaI, and the *rig-3* promoter was inserted after amplification from plasmid pCS42 using the oligos AAGCTTGGCCGGCCTTCTCTGCCTCCCTCAACTTC and AAGCTTGGCGCGCCTTTCGAAAAAGAAGAATGAAGTTCTTC. pCoS13 (p*osm-10*::loxP::LacZ::STOP::loxP:: ChR2::mCherry::SL2::GFP): Plasmid pCoS6 was cut with SphI and BamHI, and the p*osm-10* promoter inserted after amplification from plasmid pKS52 (p*osm-10*::GFP; a gift by Anne Hart, Brown University, USA) using oligos GAATTGCATGCTGCGCCTTTGAAGAGTACTG and AATTGTCGACCGAAAGTTGGCT CAACATCTC. pCS40 (p*flp-18*::FRT::mCherry::STOP::FRT::ChR2::YFP): Plasmids pCS45, pCS49, and pCS48 were conjointly used in a Gateway recombination reaction with the vector pDESTR4-R3 (Invitrogen) to generate pCS40. pCS41 (p*rig-3*::FRT::mCherry::STOP::FRT:: ChR2::YFP): Likewise, plasmids pCS46, pCS49, and pCS48 gave rise to pCS41 in a Gateway recombination reaction with vector pDESTR4-R3 (Invitrogen). pCS45 (p*flp-18* entry-Vector for Slot-1 in Gateway cloning): A ∼4,2 kbp fragment of the promoter *pflp-18* was amplified from genomic *C. elegans* DNA by PCR using oligos oCS95 (GGGGACA ACTTTGTATAGAAAAGTTGGCTCTGTCACATACTGCTCG) and oCS96 (GGGGACTGC TTTTTTGTACAAACTTGGCATGTCTAACCCTGAAA). The purified PCR product was then used in a recombination reaction with pDONR-P4-P1r and BP Clonase II (Invitrogen) to generate pCS45. pCS46 (prig-3 Entry-Vector for Slot-1 in Gateway cloning): A ∼3,1 kbp fragment of the promoter *prig-3* was amplified from genomic DNA by PCR using the oligos oCS93 (GGGGACAACTTTGTATAGAAAAGTTGGCTTCTCTGCCTCCCTCAACTTC) and oCS94 (GGGGACTGCTTTTTTGTACAAACTTGGCATTTTCGAAAAAGAAGAATGAAG). The purified PCR product was recombined with pDONR-P4-P1r using BP Clonase II (Invitrogen). pCS47: (unc-54 3′-UTR, Entry-Vector for Slot-3 in Gateway cloning): ∼0,8 kbps of the *unc-54*-UTR were PCR amplified from pAG54 [3], using oligos oCS99 (GGGGACAGCTTTCTTGT ACAAAGTGGGCTAACATCTCGCGCCCGTGCCTC) and oCS101 (GGGGACAACTTTG TATAATAAAGTTGGCGGCCGACTAGTAGGAAACAG). The purified PCR product was then used in a recombination reaction with pDONRP2R-P3 and BP Clonase II (Invitrogen) to generate pCS47. pCS48 (ChR2::YFP::unc-54-UTR, Entry-Vector for Slot-3 in Gateway cloning): A ∼2,6 kbp fragment containing ChR2::YFP::unc-54UTR was PCR amplified from pAG54 [1] using oligos oCS100 (GGGGACAGCTTTCTTGTACAAAGTGGGCGCATG GATTATGGAGGCGCCC) and oCS101 (GGGGACAACTTTGTATAATAAAGTTGGCGGC CGACTAGTAGGAAACAG). The purified PCR product was then recombined with pDONRP2R-P3. pCS49 (FRT::mCherry::STOP::FRT, Entry-Vector for Slot-2 in Gateway cloning): A ∼1,5 kbp fragment containing FRT::mCherry::STOP::FRT was PCR amplified from pWD178 [21] using oligos oCS97 (GGGGACAAGTTTGTACAAAAAAGCAGGC) and oCS98 (GGGGACCACTTTGTACAAGAAAGCTGGGTCGAAGTTCCTATACTTTCTAG). The purified PCR product was then recombined with pDONR221. pCS101 (p*flp-18*::FLP): A ∼4,2 kbp fragment of *pflp-18* was amplified from pCS45 using oligos oCS200 (GTGGATCCGCTATCAACTTTGTATAGAAAAGTTG) and oCS205 (CACAGCTAGCGT CTAACCCTGAAATTATTATTT) and cloned into pTNZ126 using BamHI and BmtI cuts. pCS102 (p*rig-3*::FLP): A ∼3,1 kbp fragment of *prig-3* was amplified from pCS46 by PCR using the oligos oCS200 (GTGGATCCGCTATCAACTTTGTATAGAAAAGTTG) and oCS203 (CACAGCTAGCTTTCGAAAAAGAAGAATGAAG). The purified PCR product was then ligated into pTNZ126 after BamHI and BmtI restricition. pCS132 (p*rig-3*::FRT::mCherry::STOP::FRT::ChR2::YFP): ∼1,5 kbp containing FRT::mCherry::STOP::FRT were amplified from pCS49 using oligos oCS229 (GTGTGCTAGCACCGGTGGGCCCGAAG TTCCTATTCTCTAGAAAG) and oCS230 (CACAGGGGCCCGAAGTTCCTATACTTTCTAG), and subsequently digested with a) AgeI and EcoRV (resulting in a 0,8 kbp fragment) and b) EcoRV and EcoO109I (resulting in 0,6 kbp fragment). In addition, a ∼2,5 kbp fragment containing ChR2::YFP::unc-54-3′-UTR was amplified from pAG54 [1] by PCR using oligos oCS231 (GTGTGGGCCCCATGGATTATGGAGGCGCCCTG) and oCS232 (GGGCCCGTAC GGCCGAC) and digested with EcoO109I and BsiWI. All three fragments were then cloned into pCS102 using the restriction enzymes AgeI and BsiWI. pCS133 (p*gpa-14b*::FLP): A ∼3,0 kbp fragment of *pgpa-14b* was amplified from genomic DNA using oligos oCS236 (GTGTCCTGCAGGACGACGACAAGAAGGTAATT) and oCS237 (CACAGCTAGCTACA CCTGAATTTTATAAG), and subcloned into pTNZ126 following SbfI and BmtI digestion. pCS134 (pglr-1::FLP): A ∼5,3 kbp fragment ofthe promoter *pglr-1* was amplified from pCS106 [33] by PCR using the oligos oCS233 (CATGCCTGCAGGGGCCGGCCGTAGCCGGTATG) and oCS234 (CACAGCTAGCC TGTGAATGTGTCAGATTGG). The purified PCR product was then cloned into pTNZ126 using SbfI and BmtI. pCS135 (p*gpa-14b*::FRT::mCherry::STOP::FRT::ChR2::YFP): ∼3,0 kbps of *pgpa-14b* were amplified from pCS133 using oligos oCS236 (GTGTCCTGCAGGACGACGACAAGAAGGTAATT) and oCS238 (CACAACC GGTTACACCTGAATTTTATAAG), and cloned into pCS132 after SbfI and AgeI digest. pCS136 (p*glr-1*::FRT::mCherry::STOP:: FRT::ChR2::YFP): A ∼5,3 kbp fragment of the promoter *pglr-1* was amplified from pCS134 using oligos oCS233 (CATGCCTGCAGGGGCC GGCCGTAGCCGGTATG) and oCS235 (CACAACCGGTCTGTGAATGTGTCAGATTGG). The purified PCR product was then cloned into pCS132 using restriction enzymes SbfI and BmtI. pSH116 (p*des-2*::Cre): Plasmid pNP259 was cut with SphI and NcoI and the *des-2* promoter inserted after amplification from genomic DNA using oligos ACGTAGCATGCGATC TCAAAGTACATACATTC and ATCCATGGCCTGTAGTAAAAGTAAATGTG.

The first 29 amino acids of the ChR2 primary structure were recognized as eukaryotic signal sequence by computational analysis (SignalP [34]) and were referred to as ChR2-signal sequence. Similarly, using an alternative upstream start codon within the genome of *Natronomonas pharaonis* added additional 19 amino acids to the amino-terminus which were recognized as eukaryotic signal peptide, again using SignalP. This sequence was cloned into plasmid pCS10: p*myo-3*::NpHR-SigSeq::NpHR::eCFP [35], and termed NpHR-signal sequence. Sites of fragmentation within ChR2(H134R) and NpHR were selected in loop-regions in order to minimize impact on functionality of the respective rhodopsin. To this end, the primary structures of ChR2(H134R) and NpHR were aligned with the homologous bacteriorhodopsin [36] and Halorhodopsin structures [37] from *Halobacterium salinarium* – using the tools ClustalW [38], HMMTOP [39], MEMSAT3 [40], and T-Coffee [41]. Furthermore, structural information about ChR2(H134R) was contributed by P. Wood and E. Bamberg and for NpHR by L. Forrest (all Max-Planck Institute for Biophysics). The topology of individual fragments was analyzed using the algorithms of TMHMM [42] and SOSUI [43]. The following plasmids were generated using standard techniques: pCS1: pmyo-3::nzYFP::ChR2 (Helices3-7; Ala111-Thr314), pCS1+: pmyo-3::ChR2-SigSeq::nzYFP::ChR2 (Helices3-7; Ala111-Thr314), pCS2: pmyo-3::ChR2 (Helices1-2; Met1-Leu110)::czCFP, pCS3: pmyo-3::nzYFP::ChR2 (Helices4-7; Asn143-Thr314), pCS4: pmyo-3::ChR2 (Helices1-3; Met1-Ser142)::czCFP, pCS5: pmyo-3::NpHR-SigSeq::NpHR::eCFP, pCS6: pmyo-3::ChR2 (Helices1-5; Met1-Gly206)::czCFP, pCS14: pmyo-3::NpHR (Helices1-3; Met1-Ser144), pCS14+: pmyo-3::NpHR-SigSeq::NpHR (Helices1-3; Met1-Ser144), pCS16: pmyo-3::NpHR (Helices 4-7; Ser144-Asp291)::eCFP, pCS20: pmyo-3::ChR2 (Helix 1, Met1-Thr74), pCS21: pmyo-3::ChR2 (Helices 1-2, Met1-Pro105), pCS22: pmyo-3::ChR2 (Helices 1-5, Met1-Gly199), pCS23: pmyo-3::ChR2 (Helices 2-7, Lys76-Thr314), pCS24: pmyo-3::ChR2 (Helices 3-7, Ser106-Thr314), pCS24+: pmyo-3::ChR2-SigSeq::ChR2 (Helices 3-7, Ser106-Thr314), pCS25: pmyo-3::ChR2 (Helices 6-7, Tyr200-Thr314), pCS26: pmyo-3::ChR2 (Helix 1, Met1-Thr74)::YFP::ChR2 (Helices 2-7, Trp75-Thr314), pCS27: pmyo-3::ChR2 (Helices 1-2, Met1-Pro105)::YFP::ChR2 (Helices 3-7, Ser106-Thr314), pCS28: pmyo-3::ChR2 (Helices 1-5, Met1-Gly199)::YFP::ChR2 (Helices 6-7, Tyr200-Thr314, pCS80: pmyo-3::NpHR (Hel. 1-2; Met1-Gly88), pCS81: pmyo-3::NpHR-SigSeq::NpHR (Hel. 1-2; Met1-Gly88), pCS82: pmyo-3::NpHR (Hel. 3-7; Leu89-Asp291)::eCFP, pCS83: pmyo-3::NpHR (Hel. 1; Met1-Pro62), pCS85: pmyo-3::NpHR (Hel. 2-7; Arg63-Asp291)::eCFP, pCS89+: pmyo-3::pat-3 SigSeq::spGFP11:: NpHR (Hel. 3-7; His100-Asp291), pCS90+: pmyo-3::NpHR (Hel. 1-2; Met1-Gly99)::spGFP1-10, pCS91+: pmyo-3::NpHR SigSeq::NpHR (Hel. 1-2; Met1-Gly99):: spGFP1-10, pCS92: pmyo-3::NpHR (Hel. 1-2; Met1-Gly99), pCS94: pmyo-3::NpHR (Hel. 3-7; His100-Asp291)::eCFP

### Fluorescence Microscopy

Expression of ChR2 with bicistronically expressed GFP using the described promoter combinations and also the ChR2::mCherry expression were analyzed on an LSM confocal laser scanning microscope, as well as on a Zeiss Axio Observer equipped with a Marianas spinning-disk confocal (SDC) system (3i - Intelligent Imaging Innovations) and an Evolve EMCCD camera (Photometrics).

### Behavioral Assays

To minimize light induced escape behavior, i.e. not mediated by ChR2, all experiments were performed in a mutant background, *lite-1(ce314)*, which is largely insensitive to blue and UV light [44], [45], and thus shows almost no negative phototaxis.

#### Response Tests

Transgenic worms were cultivated in the dark at 20°C on NGM plates with OP50-1 bacteria with or without all-*trans* retinal (ATR). Plates containing ATR were prepared by spreading 300 µl of OP50-1 culture mixed with 0.3 µl of 100 mM ATR stock (dissolved in ethanol) onto 5.5-cm plates containing 8 ml of NGM. About 18 h before the experiments, L4 larvae, grown on ATR plates, were placed on fresh ATR plates. For the measurements worms were illuminated on 5.5-cm diameter plates containing 8 ml of NGM with blue light (1.6 mW/mm^2^) from a 100-W mercury lamp, filtered through a GFP excitation filter (450–490 nm), under a 10× objective on an Axiovert 200 microscope (Zeiss, Germany). Duration of illumination was defined by a computer-controlled shutter (Sutter Instruments, USA). Every single worm was illuminated 5 times for 1 s with an ISI (interstimulus interval) of 10s and afterwards the mean of all 5 illuminations was generated. Any observable backward locomotion during or directly after (1 s) a blue light pulse was counted as a response.

#### Patterned Illumination

All worms tested were F1 progeny of P0 adults picked onto ATR plates 4 d before experiments. Young adult worms were picked onto NGM plates with a thin layer of OP50 with ATR 25 min before the experiments. Strains with non-integrated transgenes were picked on the basis of a fluorescent co-injection marker. Each animal was used only for a single experiment and then discarded. Animals were illuminated 3 times for 2 s with an ISI of 10 s and the mean of all 3 illuminations was calculated. The patterns included head region (anterior 17%), the region directly posterior of the head (17–30%) and the whole animal. The measurement and velocity analysis was conducted as described [15].

#### Analyses of contraction and relaxation

These effects were taken as indication for functional reconstitution of complementary ChR2 and NpHR fragments, respectively, were essentially performed as described previously [8]. In short, animals were recorded on non-seeded NGM plates using an Axiovert 40 CFL microscope (Zeiss) with 10× magnification and Powershot G5 or G9 digital cameras (Canon). For photoactivation, yellow light (530–560 nm; 10.2 mW/mm^2^; filter F41-007, AHF Analysetechnik) or blue light (450–490 nm; 1.6 mW/mm^2^; filter F36-525, AHF Analysetechnik) from an HBO50 light source were presented and controlled by a computer-driven shutter (Sutter Instruments). Videos were then extracted into single frames and worm length (after 560 ms photostimulation) was analyzed using a custom written script for Matlab [4] or ImageJ.

## Results

### Specific Expression of ChR2 in PVC Using the Cre-loxP System

Two genetic constructs are required to achieve single cell expression of a protein *via* this system in *C. elegans*: One encodes Cre, driven by one promoter, and the second one uses another promoter, instructing expression of the protein of interest, in this case ChR2 (**Fig. 1**). A transcriptional stop cassette (also encoding LacZ) is included between promoter and the protein of interest, flanked by loxP sites [20]. Therefore, ChR2 can only be expressed at the “intersection” of both chosen promoters. The target construct after recombination will contain the promoter, one loxP site, start codon (ATG) and coding sequence for ChR2::mCherry.

**Figure 2:**
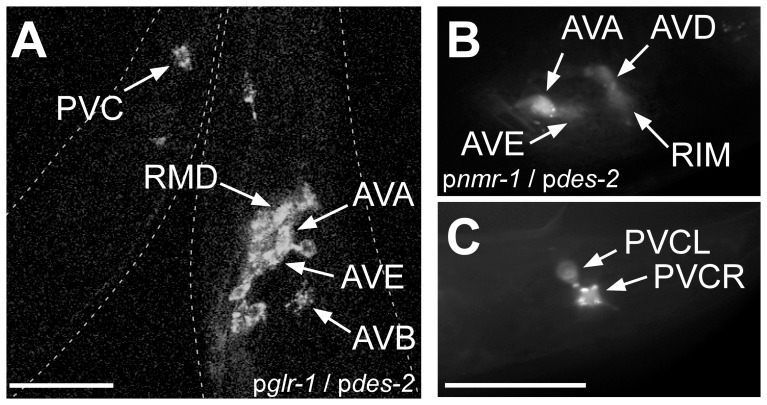
Promoter combinations chosen for expression of ChR2 in PVC neurons, using Cre recombinase, are not PVC-specific. **A**) Confocal stack of an animal expressing ChR2::mCherry in head neurons and PVC, using the *glr-1* and *des-2* promoters, resembling the p*glr-1* expression pattern. **B**) The promoter combination *nmr-1* and *des-2* led to an expression in four neurons in the head (AVA, AVB, AVD, AVE), in addition to expression in PVC in the tail (**C**). Scale bars = 20 µm.

First we tried to achieve specific ChR2 expression in the PVC command interneurons. These cells evoke forward locomotion when being photostimulated by selective illumination of the tail in a strain expressing ChR2 from the *glr-1* promoter that, however, is active in many additional neurons [46], [47]. To enable this using wide-field illumination, we attempted to express ChR2 specifically in this cell pair, for which no single-cell specific promoter is known. Promoter pairs potentially overlapping in this cell, or in any given neuron, can be deduced from the literature, i.e. as data deposited in wormbase (www.wormbase.org), and conveniently summarized for each cell in Nikhil Bathla's online tool “*C. elegans* interactive neural network” (www.wormweb.org/neuralnet#c=PVC&m=1). Based on this repository, we chose two different promoter pairs: p*glr-1*/p*des-2* and p*nmr-1*/p*des-2* (**Table 1**). Cre recombinase was placed under the control of the *des-2* promoter, while the loxP::LacZ::STOP::loxP::ChR2::mCherry construct was placed downstream of either the *glr-1* or the *nmr-1* promoter. The promoter pair p*glr-1*/p*des-2* led to an expression pattern similar to the pattern of p*glr-1* only [30] (**Fig. 2A**). The promoter pair p*nmr-1*/p*des-2* led to an expression of ChR2 in PVC in the tail but also in four additional cells in the head: AVA, AVD, AVE and RIM (**Fig. 2B, C**), i.e. essentially the expected p*nmr-1* expression pattern. The animals were not further tested in optogenetic behavioral assays, as the multitude of neurons expressing ChR2 would have caused too many cells to be activated concomitantly. PVC could be selectively photostimulated by patterned illumination of p*glr-1::ChR2* animals, leading to acceleration of the animals [46], [47]. Thus, despite predicted overlap of expression patterns in PVC, the chosen promoter combinations resulted in expression more closely resembling the *glr-1* or the *nmr-1* promoters. Possibly, expression patterns described in the literature are rather incomplete, thus Cre may be expressed in many more cells than only those described for the *des-2* promoter.

**Table 1:** Promoter combinations used for PVC specific ChR2 expression (Cre-lox system).

Promoter	Expression pattern	Combinations	
**p** ***glr-1***	AIB, AVA, AVB, AVD, AVE, AVG, AVJ, DVC, PVC, PVQ, RIG, RIM, RMD, SMD, URY [62]	*pglr-1::loxP::LacZ::STOP::loxP::ChR2::mCherry*	
**p** ***des-2***	m1 head muscles, IL2, FLP, PVD, PVC [63]	*pdes-2::Cre*	
**p** ***nmr-1***	AVA, AVE, AVG, PVC, DVA and one of: RIM, AVH, AVD, AVB [62]		*pnmr-1::loxP::LacZ::STOP:: loxP::ChR2::mCherry*
		**Expression pattern similar to p** ***glr-1*** ** pattern**	**Expression in PVC and four additional cells**

### Specific expression of ChR2 in ASH neurons

**Figure 3:**
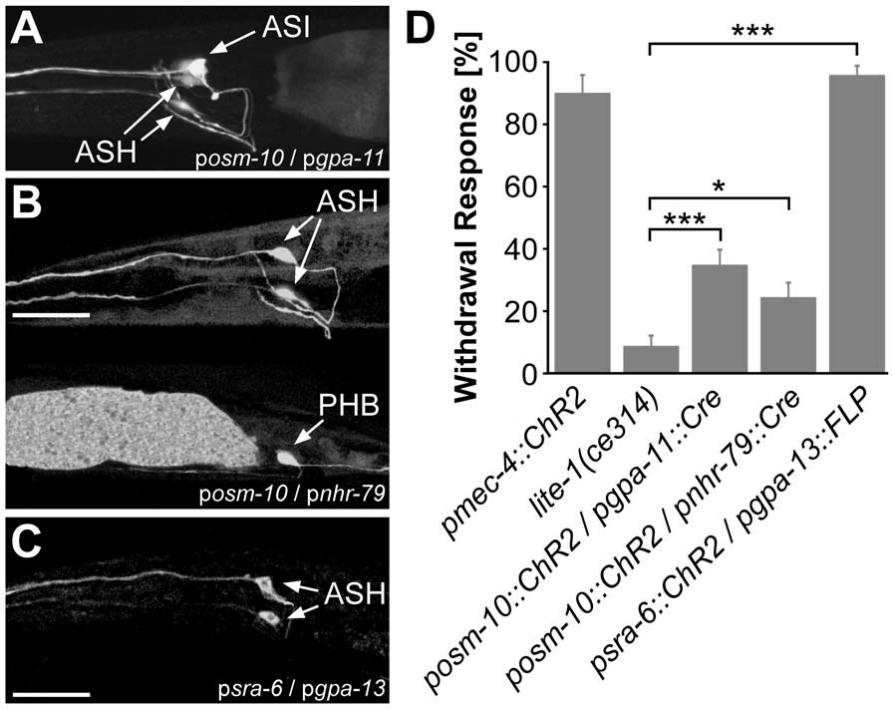
Expression pattern and behavioral responses of animals generated towards ChR2 expression in ASH by using Cre recombinase. GFP fluorescence (confocal stacks) in animals expressing ChR2::mCherry and GFP (bicistronically co-expressed) using the promoter combinations *osm-10* and *gpa-11* **A**), *osm-10* and *nhr-79* **B**), or *sra-6* and *gpa-13* **C**). Scale bar = 20 µm. **D**) Behavioral assay testing withdrawal reactions in response to 470 nm blue light illumination of the indicated conditional expression strains (all *lite-1(ce314)* background). Strain AQ2334 (pmec-4::ChR2) and *lite-1(ce314)* were used as positive and negative controls, respectively. n = 14 animals of each strain were tested, with 5 consecutive light pulses each; displayed are means ± SEM. Stastistically significant differences were determined by t-test, relative to the *lite-1(ce314)* control. * p<0.05; *** p<0.001.

To study the integration of ASH sensory neurons into the *C. elegans* nervous system, e.g. at the level of the interneurons, a possibility to specifically photostimulate ASH neurons would be desirable. We used the promoter combinations p*sra-6*/p*nhr-79*, p*osm-10*/p*gpa-11* and p*osm-10*/p*nhr-79* (**Table 2**) to achieve ChR2 expression in ASH with Cre recombinase. Here, we generated constructs that would allow expressing ChR2::mCherry, and additionally, from a bicistronic expression cassette, soluble GFP as a bright fluorescent marker, enabling to better visualize all cells likely to express ChR2. The ChR2::mCherry signal is relatively weak and clustered, thus it is difficult to unequivocally identify individual neurons *via* ChR2::mCherry only. While p*sra-6*/p*nhr-79* lines showed no visible expression, p*osm-10*/p*gpa-11* lines featured high GFP fluorescence in ASH but also a similar fluorescence in the ASI neuron (**Fig. 3A**), another amphid neuron in the head [48]. The use of the promoter pair p*osm-10*/p*nhr-79* essentially resulted in a specific expression of ChR2 in ASH, but there was also an expression in PHB, a tail phasmid neuron [49] (**Fig. 3B**). Thus, neither of these promoter pairs led to an exclusive ASH specific expression. Yet, with patterned illumination, the latter combination would allow specific ASH (or PHB) photostimulation. In behavioral assays we tested the p*osm-10*/p*gpa-11* and p*osm-10*/p*nhr-79* lines together with animals expressing ChR2 in the mechanoreceptor neurons (p*mec-4*::ChR2) as a positive control; these animals are known to reverse upon photostimulation [1]. All behavioral assays were performed in a *lite-1(ce314)* genetic background, to eliminate the intrinsic photophobic response of *C. elegans*. As a negative control, we used animals without any ChR2 expression (**Fig. 3D**). Only 35% of the p*osm-10*/p*gpa-11* and 24% of the p*osm-10*/p*nhr-79* animals responded to blue light illumination with a reversal, a value which was significantly above the negative control (8%). With the promoter pairs tested, it was thus not possible to achieve ChR2 expression which was exclusive for ASH and produced a robust blue light reaction. Meanwhile Ezcurra *et al.* established a worm strain (AQ2235) expressing ChR2 exclusively in ASH by using the FLP recombinase system with a different promoter pair, p*sra-6*/p*gpa-13* [22]. We took confocal images of these animals (a kind gift by W. Schafer) and tested them in behavioral assays (**Fig. 3C, D**). Strain AQ2235 shows a visible ChR2::YFP expression in the ASH neurons only and 98% of these animals responded with withdrawal to a blue light stimulus. We thus did not further try to optimize our own efforts to generate such animals and suggest to use AQ2235 animals in future experiments where specific ASH stimulation is needed.

**Table 2:** Promoter combinations used for ASH specific ChR2 expression (Cre-lox system).

Promoter	Expression pattern	Combinations		
**p** ***sra-6***	ASI, ASH, SPDm/SPVm, PVQ [53]	*psra-6::loxP::LacZ:: STOP::loxP::ChR2::mCherry::SL2::GFP*		
**p** ***nhr-79***	ADL, ASH [64]	*pnhr-79::Cre*		*pnhr-79::Cre*
**p** ***osm-10***	ASH, ASI, PHA, PHB [65]		*posm-10::loxP::LacZ:: STOP::loxP::ChR2::mCherry::SL2::GFP*	*posm-10::loxP::LacZ:: STOP::loxP::ChR2::mCherry::SL2::GFP*
**p** ***gpa-11***	ADL, ASH [52]		*pgpa-11::Cre*	
		**No expression**	**Expression, one additional cell pair in the head (probably ASI)**	**Expression, one additional cell pair in the tail (probably PHB)**

### Expression of ChR2 in AVA Using the FLP-System

**Figure 4:**
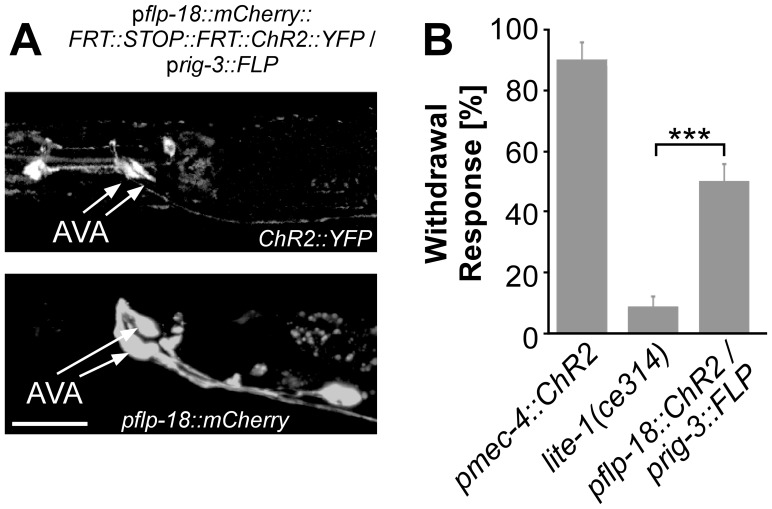
Expression pattern and behavioral responses of animals generated towards ChR2 expression in AVA by using FLP recombinase. **A**) Fluorescence images of animals expressing ChR2::YFP by using *flp-18* and *rig-3* promoter pair with the FLP recombinase (top). Bottom panel shows p*flp-18*::mCherry expression. Scale bar = 20 µm **B**) Behavioral assay testing withdrawal reactions in response to 470 nm blue light illumination were as described in Fig. 3D (n≥15).

We next tested conditions allowing expression of ChR2 specifically in AVA backward command neurons using either the FLP or the Cre (see below) recombinase systems. To express ChR2 in AVA using FLP recombinase, we used the promoter pairs p*glr-1*/p*gpa-14*, p*gpa-14*/pglr-1, p*rig-3*/p*flp-18*, and p*flp-18*/p*rig-3* (**Table 3**). We first used Gateway cloning, as for the constructs originally described by Davis *et al.* (2008) [21]. However, this results in additionally translated sequence that can interfere with the function of the proteins expressed. Thus, we also used conventional cloning techniques, which in our hands led to better results. We also added individual start codons for each mCherry and ChR2::YFP. Only when we used conventional cloning, and only with one combination of promoters, we observed notable expression of ChR2::YFP in AVA, i.e. p*flp-18::ChR2::YFP*/p*rig-3::FLP* (**Fig. 4A**). cDNA encoding mCherry was inserted in the stop cassette, based on the constructs by Davis *et al.* (2008) [21], such that cells in which p*flp-18* is expressed, and in which FLP recombinase was not active, showed red fluorescence (**Fig. 4A**, bottom). In contrast, FLP recombinase expression activated ChR2::YFP expression, leading to visible YFP fluorescence, in AVA, but also in other cells, most likely M2 and RIM (**Fig. 4A**, top). Subsequently, we tested the withdrawal reaction of these animals to a blue light stimulus. The *pflp-18::ChR2::YFP*/p*rig-3::FLP* line showed withdrawal behavior in 49% of the animals tested (**Fig. 4B**). However, as additional cells expressed ChR2::YFP in these animals, we cannot conclude that the behavior was purely evoked by AVA neurons. As the other lines did not visibly express ChR2, we did not test them in behavioral assays.

**Table 3:** Promoter combinations used for AVA specific ChR2 expression (FLP system).

Promoter	Expression pattern	Combinations			
**p** ***glr-1***	AIB, AVA, AVB, AVD, AVE, AVG, AVJ, DVC, PVC, PVQ, RIG, RIM, RMD, SMD, PVQ, URY [62]	*pglr-1::FRT::mCherry::STOP::FRT::ChR2::YFP*	*pglr-1::flp*		
**p** ***gpa-14***	ASI, ASJ, ASH, ASK, ADE, PHA, PHB, ALA, AVA, CAN, DVA, PVQ, RIA, vulva muscles [52]	*pgpa-14::flp*	*pgpa-14::FRT::mCherry::STOP:: FRT::ChR2::YFP*		
**p** ***flp-18***	AVA, AIY, RIG, RIM, M2, M3 [66]			*pflp-18::FRT:: mCherry::STOP:: FRT::ChR2::YFP*	*pflp-18::flp*
**p** ***rig-3***	AVA, I1, I4, M4, NSM, amphid sheath cells [54]			*prig-3::flp*	*prig-3::FRT:: mCherry::STOP:: FRT::ChR2::YFP*
		**no expression**	**no expression**	**weak in AVA and M3, RIM**	**no expression**

### Specific expression of ChR2 in AVA neurons using the Cre-loxP System

**Figure 5:**
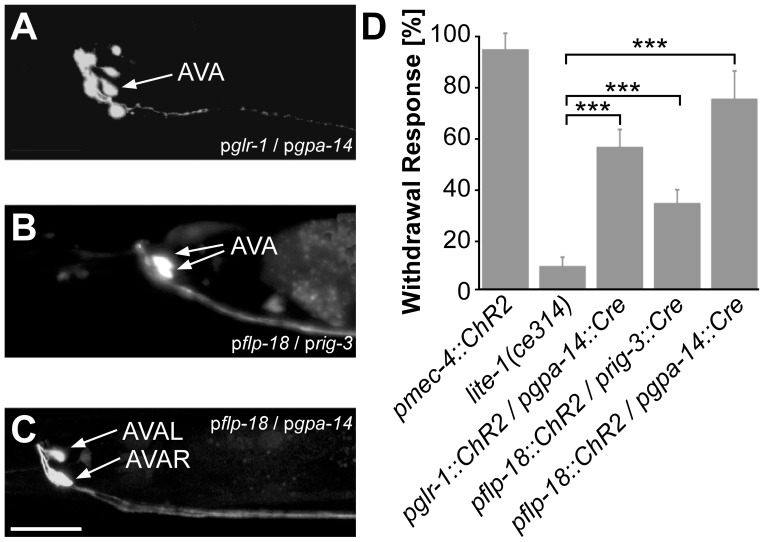
Expression pattern and behavioral responses of animals generated towards ChR2 expression in AVA by using Cre recombinase. GFP fluorescence (confocal stacks) in animals expressing ChR2::mCherry and GFP (bicistronically co-expressed) using the promoter combinations *glr-1* and g*pa-14* **A**), *flp-18* and *rig-3* **B**), or *flp-18* and *gpa-14* **C**). Scale bar = 20 µm. **D**) Behavioral assay testing withdrawal reactions in response to 470 nm blue light illumination were as described in Fig. 3D (n = 14).

As we could not achieve specific expression in AVA using the promoters described and FLP recombinase, we turned to the Cre recombinase and an additional promoter combination. Accordingly, we tested these three promoter pairs: p*glr-1*/p*gpa-14*, p*flp-18*/p*rig-3*, and p*flp-18*/p*gpa-14* (**Table 4**). To better visualize the cells, ChR2::mCherry was again linked to GFP in a bicistronic cassette. The combination of p*glr-1* and p*gpa-14* led to an expression in 10 different cells and was thus not specific for AVA (**Fig. 5A**). The second combination tested (p*flp-18*/p*rig-3*) showed an expression of GFP in AVA and also a minor expression in AIY, a pair of neurons which is involved in thermosensation [50]. Finally, the combination of p*flp-18* and p*gpa-14* showed bright expression of GFP in AVA neurons only (**Fig. 5C**). We carried out behavioral assays to test the reaction of the three Cre-loxP AVA lines to blue light illumination (**Fig. 5D**). As a positive control we used animals expressing ChR2 in touch neurons (p*mec-4*::ChR2). As a negative control, we used *lite-1(ce314)* animals expressing no ChR2. 93% of the positive control nematodes showed a backward movement, i.e. full reversal, not just slowing of forward locomotion, during or directly after a 1 s blue light stimulus, while the Cre-loxP lines showed a reaction between 33% and 73%. The highest reaction was observed in the line with the p*flp-18*/p*gpa-14* promoter pair, i.e. the one with the AVA specific ChR2 expression.

**Table 4:** Promoter combinations used for AVA specific ChR2 expression (Cre-lox system).

Promoter	Expression pattern	Combinations		
**p** ***glr-1***	AIB, AVA, AVB, AVD, AVE, AVG, AVJ, DVC, PVC, PVQ, RIG, RIM, RMD, SMD, PVQ, URY [62]	*pglr-1::loxP::LacZ:: STOP::loxP:: ChR2::mCherry:: SL2::GFP*		
**p** ***gpa-14***	ASI, ASJ, ASH, ASK, ADE, PHA, PHB, ALA, AVA, CAN, DVA, PVQ, RIA and vulva muscle cells [52]	*pgpa-14::Cre*		*pgpa-14::Cre*
**p** ***flp-18***	AVA, AIY, RIG, RIM, M2, M3 [66]		*pflp-18::loxP::LacZ::STOP::loxP:: ChR2::mCherry::SL2::GFP*	*pflp-18::loxP::LacZ::STOP::loxP:: ChR2::mCherry::SL2::GFP*
**p** ***rig-3***	AVA, I1, I4, M4, NSM and amphid sheath cells [54]		*prig-3::Cre*	
		**Expression in AVA, but also in 8 additional cells**	**Expression in AVA, but also in additional cells (probably M3, AIY, RIM)**	**Single cell expression in AVA; low expression in RIG following integration**

**Figure 6:**
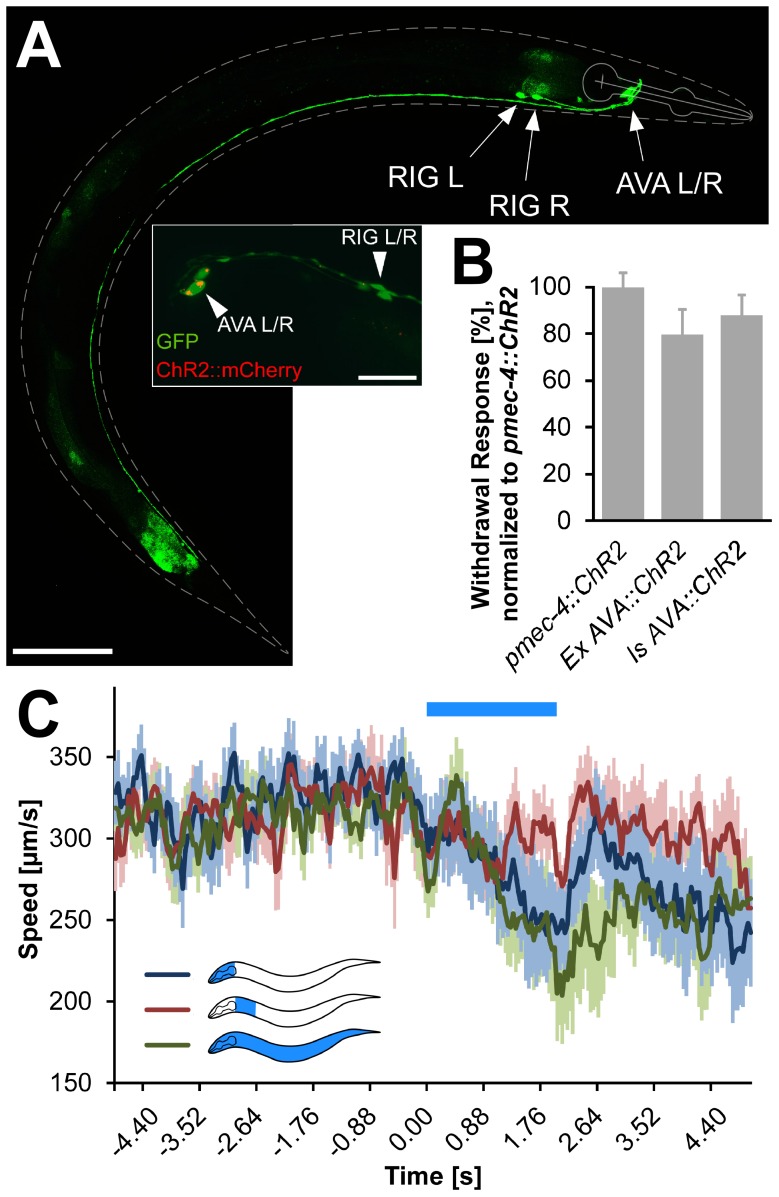
ChR2::mCherry::SL2::GFP expression in AVA (and RIG) neurons using *flp-18* and *gpa-14* promoters and Cre recombinase from an integrated transgene. **A**) Confocal stack showing GFP fluorescence of the specific expression pattern. Scale bar = 100 µm (20 µm in inset). Inset: Overlay of the ChR2::mCherry (red) and the much brighter SL2::GFP (green) expression. **B**) Withdrawal in response to whole-animal blue illumination, compared in the transgenic line before (Ex) and after chromosomal integration (Is) was compared after normalization to the responses of p*mec-4*::ChR2 animals tested alongside. **C**) Locomotion speed traces of animals of the integrated strain expressing ChR2 in AVA (and RIG) before, during and after patterned illumination of either of two different segments in the head region (blue, segment harboring AVA cell bodies; red, segment harboring RIG cell bodies, and parts of the AVA axons). In addition, the whole animal was illuminated (green). Displayed are means ± SEM (n≥15 in B and C). Duration of the light stimulus is indicated by a blue bar.

To consolidate the blue light reaction of the p*flp-18*/p*gpa-14* animals, we integrated the extrachromosomal array into the genome. The expression pattern of the bicistronic GFP (**Fig. 6A**) again featured fluorescence in AVA neurons. However, we now observed expression also in the RIG neuron pair [26] (C. Bargmann, personal communication). RIG neurons are a pair of interneurons localized in the retrovesicular ganglion, and involved in reversal behavior [51]. Thus, whole body illumination to activate ChR2, which evoked reversals in ca. 80% of the animals tested (**Fig. 6B**), may affect behavior *via* RIG as well. However, GFP expression in RIG was much lower compared to AVA, and in confocal z-projections, we found no noteworthy expression of ChR2::mCherry in RIG neurons (**Fig. 6A**, inset). Thus, the ChR2 expression level in RIG is likely too weak to cause blue light-evoked behavior. To demonstrate this, we carried out behavioral assays using selective illumination of the nematode, enabling photostimulation of AVA or RIG cell bodies separately (**Fig. 6C**). The integrated strain ZX1023 p*flp-18*/p*gpa-14* responded with a decrease of velocity following blue light exposure (470 nm) of either the body segment harboring AVA neurons only, or to the whole body. Importantly, there were no marked changes in velocity following illumination of the segment harboring RIG neurons (**Fig. 6C**). Note that for this strain, light intensities achievable by the tracking and illumination system used did not allow to induce full reversal behavior to be evoked, while ZX1023 animals clearly responded with full reversals when using a different microscope (**Fig. 6B**). We conclude that ChR2 expression in RIG is too low to evoke obvious behavioral effects after illumination, and that strain ZX1023 can be used for AVA-specific neuronal photoactivation.

## Discussion

Optogenetic approaches in *C. elegans* would largely benefit from generic methods that allow expression of optogenetic tools in single cells, such that straightforward whole-field illumination can be used to stimulate just the cell of interest. Such methods, based on conditional expression at the intersection of two promoters, have been realized for *C. elegans* for GFP or other proteins, using FLP or Cre recombinases [20]–[22]. In the present study, we used both systems for expression of ChR2 in several sensory neurons or interneurons. We thus established functional “single” cell expression of ChR2 in the AVA neuron pair using the promoters for *flp-18* and *gpa-14*. Additionally, we tested several promoter combinations targeting PVC as well as ASH neurons, but these attempts were only partially successful, mainly due to the apparently incomplete promoter expression patterns reported in the literature. Nevertheless, single-cell expression in ASH was demonstrated by Ezcurra *et al.* (2011) [22], using the p*sra-6*/p*gpa-13* promoter combination, and FLP recombinase. We tested these animals in functional assays, comparing them to other promoter combinations targeting ASH, or single promoter expression via *sra-6*. Depending on the promoter combination used, and on relative expression levels of recombinase and ChR2 construct, both FLP and Cre systems allowed the generation of useful transgenic lines. In essence, both methods are very useful, however, considerable effort in empirically determining the right promoter combination, and in optimizing expression conditions, may be required.

The most substantial challenge in any of the conditional expression approaches is to find promoter pairs suited for true cell specific expression. Most expression patterns reported in the literature depend on transcriptional promoter-fluorescent protein fusions, or full-length tagged fusion proteins, some also on antibody staining [52]–[54]. Fusion proteins usually produce less fluorescent signal than expression of just GFP [55]–[57], and thus it can be expected that often cells are overlooked or not reported if they are only weakly expressing the reporter. It is difficult to judge this from published work, as often fluorescence is shown from a single focal plane or a select region of the animal [52], making it impossible to estimate comprehensively the expression pattern of a particular promoter fragment. Expression patterns may also change if a given promoter is combined with different coding sequences, due to possible or cryptic enhancers present in one but not the other cDNA. Lastly, identifying *C. elegans* neurons unequivocally is not a trivial task, so it can be expected that some neurons are wrongly assigned for a given promoter. Thus, the most prevalent way of improvement would be to have more complete and correct expression patterns for *C. elegans* promoters, or, ideally, promoter combinations in conditional expression approaches, as it was pioneered by Zhang *et al.* (2004) [17]. However, even this information would not guarantee that expression patterns established this way would be identical if used to express optogenetic tools. Further manipulation e.g. by genomic integration of the multicopy tandem array, may alter the achieved expression patterns, as we observed for expression in AVA: High(er) expression of Cre may cause unwanted recombination between distal loxP sites in the tandem array, potentially bringing ChR2 coding sequence close to a cryptic enhancer sequence, leading to expression in other cells. In order to better estimate the finally achieved expression pattern, we used a SL2-bicistronically expressed GFP, downstream of ChR2::mCherry, leading to strong GFP expression throughout the cytosol, allowing to detect even cells with weak expression patterns (**Fig. 6A**), as described by Macosco et al. (2009) [20]. In the case of the RIG neuron, which was well visible by GFP expression, expression of ChR2::mCherry was hardly detectable and blue-light activation by selective illumination was so low that no appreciable influence on behavior could be observed.

Another point to consider is which of the respective two promoters is used to drive ChR2 expression, and which one for the recombinase gene. For example, we observed expression of ChR2::YFP in the p*flp-18*::FRT::mCherry::STOP::FRT::ChR2::YFP + p*rig-3*::FLP animals, but not in the animals of the opposite combination. Promoter strength influences the efficiency of expression, and in most cases, it will be desired to achieve as much ChR2 expression as possible. Therefore, it is favorable to choose the stronger promoter for ChR2 and the weaker one for the respective recombinase. The relative amounts of injected DNA for ChR2 and recombinase construct may also require optimization, as this is affecting relative expression levels of the two transgenes.

The mode of operation of both recombinase systems used (FLP and Cre) is analogous [21], [24]. Some authors reported that the efficiency of both systems in cultured cells and in mice is comparable [58]–[60], while others showed less efficiency of the FLP system on chromosomal targets [61] and in murine embryonic stem cells. In our experiments, FLP recombinase caused no observable expression of ChR2::YFP for most of the promoter combinations tested, except for the p*flp-18* and p*rig-3* combination. However, behavioral assays were done with these animals. In comparison to the analogous animals using the Cre recombinase system, this demonstrated a significantly more frequent blue light reaction of the animals transformed with the FLP system – while these animals expressed ChR2 in cells in addition to AVA. Yet, a different promoter combination, and using the Cre system, generated more robustly responding animals expressing ChR2 in AVA neurons. Thus, it depends on the promoter combination which system is the more efficient one.

We could demonstrate that it is generally possible to establish a neuron specific ChR2 expression by application of FLP or Cre recombinase. This significantly expands the possibilities for neuroscience research in *C. elegans*, as many more neurons should now become accessible to single-neuron optogenetic manipulations. Possibly, if more labs adopt these techniques, the joint effort of the *C. elegans* researcher community may in the long run generate a set of animals (or, at least, tested promoter combinations) for essentially every neuron of *C. elegans*. This would allow generating or combining strains with different optogenetic actuators (ChR2, halorhodopsin, proton pumps like Mac or Arch, as well as color-shifted ChR chimeras like C1V1 [6]) in individual cells of a given neuronal circuit under study. These could be used to precisely probe the function of each neuron in the generation of particular behaviors, or in network function, when using Ca^2+^ imaging as a readout [47]. Furthermore, even if single-cell expression cannot be achieved in all cases, use of multimodal selective illumination technology for freely behaving animals, as recently introduced [15], [16], [46], may allow to achieve single neuron activation, provided that the neurons expressing ChR2 are located sufficiently far apart in the animal.

## Supporting Information

Figure S1 Fragment reconstitution of genetically split ChR2 and NpHR opsins in body wall muscle cells. ChR2 and NpHR were genetically split and resulting N- and C-terminal fragments were separately co-expressed in body wall muscle cells. Photoactivation with either blue (for ChR2 fragments) or yellow light (for NpHR fragments) was applied and resulting behavioral effects (contraction or relaxation) were measured and compared to the effects evoked by the respective full-length opsin to test functional reconstitution. **A, B**) Schematics of ChR2 (A) and NpHR (B), depicting heptahelical topology and sites of fragmentation, or YFP insertion, as indicated by colored arrowheads. Aminoacids flanking the fragmentation sites are given and contractions (ChR2) or relaxations (NpHR), respectively, for co-expression and photostimulation of complementary fragments or full-length opsins are indicated. Optionally, either the spGFP (green arrowheads; [32]) or cCFP/nYFP system (yellow arrowheads; [17]) were applied. When no split fluorophore was used (red arrowheads), eCFP was added to the C-terminus of NpHR. Where applicable, putative signal sequences (“sigseq”; aa 1–27 of ChR2 or aa -19-0 of NpHR [35] were added to the C- (ChR2) or N-terminal halves (NpHR), respectively, to ensure proper expression and membrane topology. **C**) Schematic depicting the arrangement of split-fluorophores that were optionally added to some fragmentation sites to visualize and also enforce reconstitution of fragments (indicated with green and yellow arrowheads in A and B). Using GFP as described in [32], helices 1–10 were coupled to the C-terminus of N-terminal opsin fragments and helix 11 was added to the N-terminus of C-terminal fragments. Alternatively, a C-terminal fragment of CFP and an N-terminal fragment of YFP [17] were fused to the C- and N-terminal opsin fragments via antiparallel leucine zippers. **D, E**) Photoactivation with either blue (for ChR2 fragments, D) or yellow light (for NpHR fragments, E) resulted in behavioral effects (contraction or relaxation) as measured and compared to the respective full-length opsin to test functional reconstitution. Displayed are changes in the relative bodylength after 560 ms of photostimulation; aa = aminoacid, n.d. = not determined.
(TIF)
