# Correction: Specific Expression of Channelrhodopsin-2 in Single Neurons of *Caenorhabditis elegans*

**DOI:** 10.1371/annotation/7983e1b9-09e4-4123-b1d5-aaaa0121e76a

**Published:** 2013-02-15

**Authors:** Cornelia Schmitt, Christian Schultheis, Navin Pokala, Steven J. Husson, Jana F. Liewald, Cornelia I. Bargmann, Alexander Gottschalk

Navin Pokala and Cornelia I. Bargmann were erroneously omitted from the author list. The correct byline is:

Cornelia Schmitt^1,2^, Christian Schultheis^1,2^, Navin Pokala^3^, Steven J. Husson^1,2,4^, Jana F. Liewald^1,2^, Cornelia I. Bargmann^3^, Alexander Gottschalk^1,2*^

**1** Buchmann Institute for Molecular Life Sciences, Goethe-University, Frankfurt, Germany, **2** Institute of Biochemistry, Goethe-University, Frankfurt, Germany, **3** Howard Hughes Medical Institute, Laboratory of Neural Circuits and Behavior, The Rockefeller University, New York, USA, **4** Functional Genomics and Proteomics, Katholieke Universiteit, Leuven, Belgium

The correct citation is:

Schmitt C, Schultheis C, Pokala N, Husson SJ, Liewald JF (2012) Specific Expression of Channelrhodopsin-2 in Single Neurons of Caenorhabditis elegans. PLoS ONE 7(8): e43164. doi:10.1371/journal.pone.0043164

NP and CIB

designed research, provided reagents and analyzed the data.

The acknowledgments section should read as: We thank A. Hart, G. Jansen, E. Jorgensen, M. de Bono, M. Chalfie, and W. Schafer for plasmids and strains, and J. Stirman and H. Lu for help with the tracking and illumination system. We are indebted to P. Wood, L. Forrest, and E. Bamberg for help on evaluating structural and topological featuresof ChR2 and NpHR and derived fragments.

In the Plasmids section of Materials and Methods, the first sentence should read: The following plasmids used: pNP165: P*glr-1*::flox::ChR2::mCherry, pNP259: P*gpa-14*::Cre, pNP260: P*nmr-1*::flox::ChR2::mCherry.

