# Supplementary figures and images for: Specific Expression of Channelrhodopsin-2 in Single Neurons of Caenorhabditis elegans
Source: PLoS One. 2012 Aug 30;7(8):e43164. doi: 10.1371/journal.pone.0043164 (PMC3431400; doi:10.1371/journal.pone.0043164)

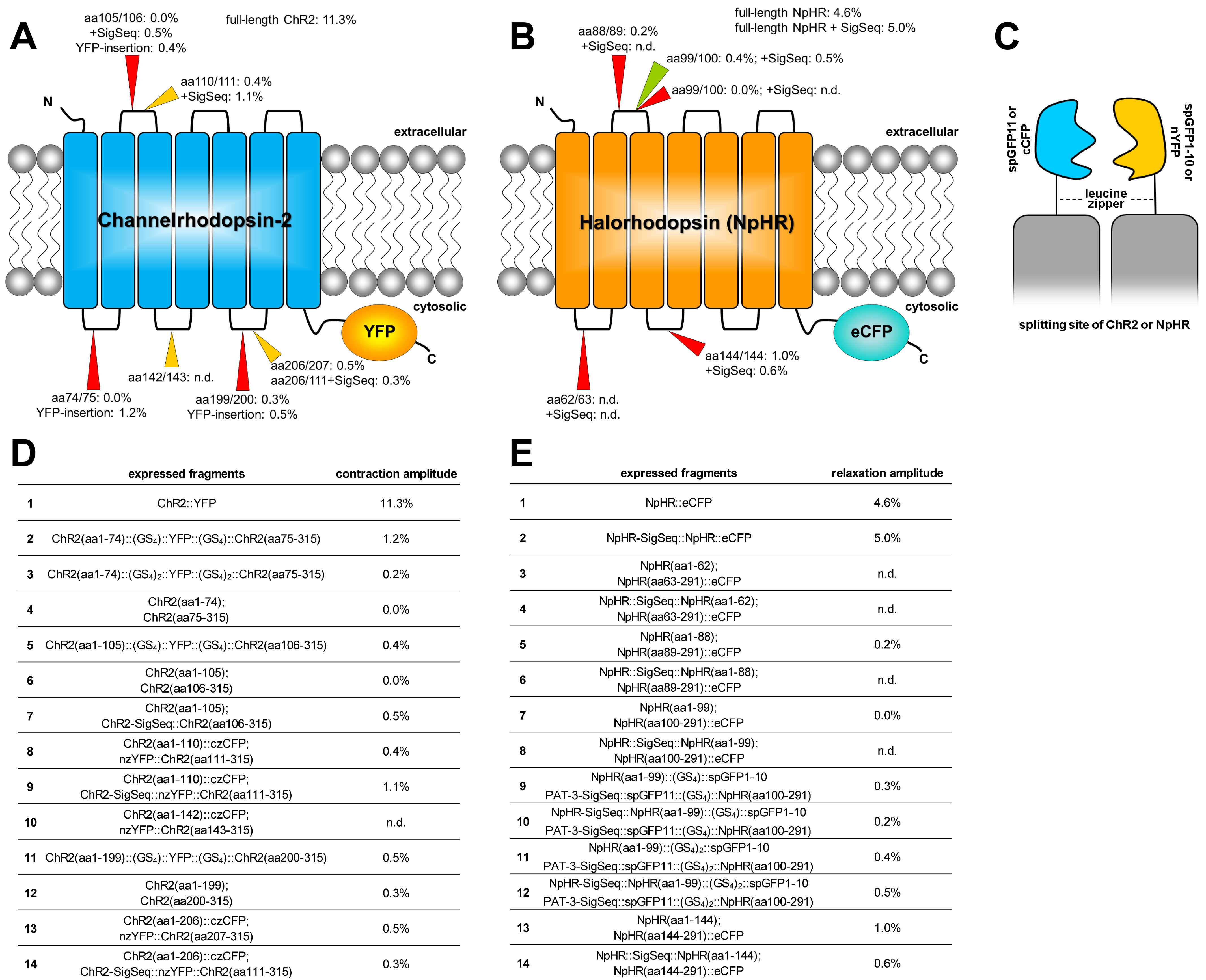

Supplement: Figure S1 — Fragment reconstitution of genetically split ChR2 and NpHR opsins in body wall muscle cells. ChR2 and NpHR were genetically split and resulting N- and C-terminal fragments were separately co-expressed in body wall muscle cells. Photoactivation with either blue (for ChR2 fragments) or yellow light (for NpHR fragments) was applied and resulting behavioral effects (contraction or relaxation) were measured and compared to the effects evoked by the respective full-length opsin to test functional reconstitution. A, B) Schematics of ChR2 (A) and NpHR (B), depicting heptahelical topology and sites of fragmentation, or YFP insertion, as indicated by colored arrowheads. Aminoacids flanking the fragmentation sites are given and contractions (ChR2) or relaxations (NpHR), respectively, for co-expression and photostimulation of complementary fragments or full-length opsins are indicated. Optionally, either the spGFP (green arrowheads; [32]) or cCFP/nYFP system (yellow arrowheads; [17]) were applied. When no split fluorophore was used (red arrowheads), eCFP was added to the C-terminus of NpHR. Where applicable, putative signal sequences (“sigseq”; aa 1–27 of ChR2 or aa -19-0 of NpHR [35] were added to the C- (ChR2) or N-terminal halves (NpHR), respectively, to ensure proper expression and membrane topology. C) Schematic depicting the arrangement of split-fluorophores that were optionally added to some fragmentation sites to visualize and also enforce reconstitution of fragments (indicated with green and yellow arrowheads in A and B). Using GFP as described in [32], helices 1–10 were coupled to the C-terminus of N-terminal opsin fragments and helix 11 was added to the N-terminus of C-terminal fragments. Alternatively, a C-terminal fragment of CFP and an N-terminal fragment of YFP [17] were fused to the C- and N-terminal opsin fragments via antiparallel leucine zippers. D, E) Photoactivation with either blue (for ChR2 fragments, D) or yellow light (for NpHR fragments, E) resulted in [file pone.0043164.s001.tif]
